# *It Can’t Happen Here* (Or Can It?) *The Deadly Rise of Anti-Science—A Scientist’s Warning* A Conversation with Author Peter J. Hotez

**DOI:** 10.4269/ajtmh.24-0292

**Published:** 2024-05-28

**Authors:** Claire Panosian Dunavan

**Affiliations:** UCLA Division of Infectious Diseases 911 Broxton Avenue, Suite 301 Los Angeles, California E-mail: cpanosian@mednet.ucla.edu

Well before our recent pandemic, few if any American scientists were more passionate and public than Peter J. Hotez, MD, PhD in countering modern misconceptions about vaccines. Then, in 2018, the prolific writer got personal. In a book called “*Vaccines Didn’t Cause Rachel’s Autism*,”^1^ Hotez not only debunked mistruths and conspiracy theories but also shared his family’s story and scientific facts about his (now-adult) daughter’s diagnosis.

Enter the virulent rhetoric and misinformation spawned by the Covid-19 pandemic. In “*The Deadly Rise of Anti-Science—A Scientist’s Warning*,”^2^ Hotez’s latest book released in 2023, ASTMH’s past-president (who is also Dean of the National School of Tropical Medicine and a Professor of Pediatrics, Molecular Virology, and Microbiology at Baylor College of Medicine) ranges even wider. In chapters with titles like “An Army of Patriots Turns Against the Scientists,” “Health Freedom Propagandists,” and “Red Covid,” Hotez juxtaposes recent political events and actors with the deadly human cost of disinformation fueled by modern media and internet algorithms. He also presents historical examples and undeniable hints of an emerging, global “ecosystem” that could quash scientific progress. And, once again, there’s the powerful element of personal story foreshadowed in an opening dedication to “the world’s scientists under threat from authoritarian leaders who seek to intimidate us” coupled with appreciation for police and security personnel at Texas Medical Center, the Texas Children’s Hospital, and Baylor for “keeping my family safe” and the Anti-Defamation League Southwest for “advice and help on rising anti-Semitic attacks and aggression.”

Of course, there’s much more about Hotez that makes him relatable to ASTMH members and friends of every background and belief. Key benchmarks include his early fascination with science; his career focus on impoverished people and neglected tropical diseases; and his lifelong goal of making a hookworm vaccine. Finally, there’s his recent pivot to developing (with Maria Elena Botazzi and other collaborators in Houston) the patent-free Covid-19 vaccine technology produced by Biological E in India and called Corbevax, and in Indonesia (where it was produced by BioFarma and called IndoVac and also designated as Halal) which has now been given to “millions of people” in low- and middle-income countries as stated in a 2024 profile in *The Lancet* titled “Peter Hotez: physician-scientist-warrior combating anti-science.”^3^^,^^4^

Nonetheless, what stood out most starkly to me in Hotez’s new book was his genuine fear that ignorance, passivity, and partisan politics could soon trigger a truly dark era in science as we currently know it—a period that both halts progress and diminishes what has already been achieved by researchers and professionals committed to human health. Could it really happen here? I wondered as I read. Given the stakes, this question is no longer a reflex response to the title of Sinclair Lewis’s long-ago, politically-prescient novel,^†^ “*It Can’t Happen Here,”*^5^ but something highly relevant to today.

Bottom line: as Hotez skillfully argues in “*The Deadly Rise of Anti-Science*,”^2^ today’s signposts are way too worrisome to ignore.

So now I’ll add my own conviction. Yes, it could happen here. Accordingly, this book—the work of a fearless colleague and author with irrepressible intellect, provocative ideas, and commendable solutions—underscores issues we must all confront and discuss. And not just with each other, but in new settings that are sometimes far beyond our usual zones of comfort.

What follows is an interview with Hotez that was initially conducted in February 2024 and subsequently edited for clarity and length.

## INTERVIEW WITH DR. PETER J. HOTEZ

**Peter, let’s jump right in. The usual stance of scientists is to remain publicly neutral, especially when it comes to political issues. In light of the rising tide of anti-science, do you believe this must now change?** I think it will have to because the attacks are so partisan. No one in science wants to talk about politics, liberals and conservatives, Republicans and Democrats, red and blue states. Our training says we should be politically neutral, and I support that. But what do you do when the 200,000 needless deaths due to refusing Covid vaccinations overwhelmingly occur in red states? In addition, as the data show: the redder the county, the lower the immunization rate, and the higher the death rate.

It’s not that we really care about people’s politics. But how do we as scientists uncouple the anti-science attacks from politics? After all, everyone is entitled to their political views–even extreme political views. That’s our right as American citizens. But how do we uncouple the anti-science because it doesn’t belong and it’s going to kill people?

There’s a comment I like from a Pugwash Conference^‡^ on Science and World Affairs in which someone who won the Nobel Prize for nuclear disarmament said that the idea that science is politically neutral shattered with the Hiroshima bomb. I think there’s truth to that, and we have to start thinking in those terms and talk about politics in order to solve problems.

**To solve problems, don’t we also need to understand different drivers of anti-science attitudes? For example, isn’t religion more of a factor in the U.S. than in Europe? Or are previous regional differences beginning to erode?** Well, we’re now seeing a shift in former differences. I would often talk to Heiden Larson,^§^ a professor of medical anthropology at the London School of Hygiene and Tropical Medicine who for years has run the Vaccine Confidence Project in London. For years, we would see each other at meetings or talk on Zoom like you and I are doing now, but we never quite meshed in terms of what she was describing in France and elsewhere in Europe, where they had their own unique flavors of anti-vaccine activism. And of course, in Afghanistan and Pakistan, it was about the Taliban and assassinating polio workers. It was not that Heidi and I disagreed; it was just that the forces driving anti-vaccine activism in the U.S. were so different from elsewhere.

Now, not so much. Now there’s more of a convergence and we’re all starting to see U.S.-style anti-vaccine activism on the far right in other parts of the world. You’re also seeing it on the far right in Germany and Austria, and the same thing in France. It’s become a new signature of political extremism and authoritarianism as shown by Jair Bolsonaro in Brazil and Viktor Orban in Hungary. This is the same thing we saw with Stalin in the 1930s when he was targeting science and scientists.

**Please talk about an organization meant to fight anti-science that you likened in your book to the Southern Poverty Law Center. Isn’t there a risk this could be seen as another bastion of left-leaning elites?** The idea behind it came from climate scientists when they realized that they were under threat and the attacks on them were highly partisan. In other words, the partisan attacks were not only seeking to discredit science, but also portraying scientists as public enemies. Government scientists were especially vulnerable if they were under political attack without the backing of traditional scientific societies. So, the climate science community organized to create a climate science defense fund that’s been around now for a few years. It’s not that big; I think it only has two full-time lawyers. But if people are sending you letters on legal stationery and you don’t know where to turn because you don’t have the backing of your university, this is a good organization to have.

Should we create something similar for biomedical scientists? In theory, we shouldn’t have to because of all the scientific societies and government organizations who should be out there defending us, but the truth is they’re often silent. ASTMH has been an exception and a wonderful source of support. I’m now in conversations about creating this kind of organization.

**Okay. Now let’s step back and talk about big-picture priorities within the biomedical community in response to anti-science.** The way I see it there are three circles of the Venn diagram that partially overlap and reflect three leading needs. One, we need to combat disinformation. This may be the biggest challenge of all–it’s a very tough one. Then there’s improving science communication, which you and I have both been working on for a long time. But now there’s a third one: defending the scientists. People conflate the three circles but they’re not completely the same.

So, here’s the monster problem: how do we approach these three circles. Bill Novelli,^‖^ Reed Tuckson^¶^ and others have organized a new consortium organization, the Coalition for Trust in Health and Science.^#^ I think it’s going to be looking at how to combat disinformation and improve science communication, but it’s still not working on defending the individual scientist. We’re working on getting further funding and seeing how to create something like that. If our societies were willing to step up, we wouldn’t need it. ASTMH has been better than most, and highly supportive of me personally (and I’m grateful for that!), but we’re just going to need more of it, even though it takes us at time into politics, which takes us out of our comfort zone. Nobody wants to talk about partisan politics, but this may become a new reality.

Meanwhile, what’s happening right now from several members of the U.S. Senate and the U.S. House of Representatives is really worrisome. Rather than have some accountability around the 200,000 Americans who perished because they refused a Covid vaccine, now they’re doubling down. They’re saying: “No, no, it was the vaccines that actually killed Americans,” which is ridiculous. And they’re saying that the scientists, the virologists, invented the virus, which is absolute nonsense.

This includes troubling actions by a House subcommittee on COVID. On their official Twitter site,** the subcommittee even says: “Get your popcorn ready folks…” I mean, so they’re not even pretending this is anything other than news soundbites and political theater. This could be very damaging, and we’ll have to see what happens after the presidential election in November. Who controls the House? Who controls the Senate? It’s already been mentioned that certain people want to dismantle the National Institute of Allergy and Infectious Diseases (NIAID), the major NIH institute that supports ASTMH members, and break it up like Ma Bell into three different units. Some policymakers want to defund infectious diseases research and outlaw certain vaccine research.^6^ These are very dark times, and if we are passive and don’t say something, then these new partisan factions could have their way.

**What about certain pro-science folks, including physicians, who are not researchers? What would persuade them to engage in this discourse?** You’re right. Most of us, whether we’re on the left or the right, signed up for this because we wanted to be physicians, physician-scientists, scientists, or public health practitioners. We want to focus on our work and our patients. We care. We went into science for humanitarian reasons, although most scientists don’t articulate that, and people have different levels of tolerance for stepping up and getting involved.

I think where you’re really going to see a wider response is if the politicians or elected leaders start taking chunks out of the NIH and NSF budgets to the point where progress stops, or they put in such onerous rules around excessive biosafety requirements or documentation to a point that research is hindered. While there is always room for improvement, in my opinion the community of virologists in the U.S. has done a good job of oversight and concern for biosafety. With continued pressure, I’m concerned you’ll start to see a brain drain from the U.S. to Canada or Europe or that sort of thing.

**Let’s discuss additional solutions.** First of all, I don’t think we really know what to do, partly because it’s a political problem. For example, you’re seeing the U.S. Surgeon General, Dr. Vivek Murthy, working with the social media companies in order to prevent them from promoting health disinformation. That was a good thing to do. But then he had to face getting sued by the Missouri Attorney General^7^ for trying to stop disinformation. That’s a problem.

Another approach is to examine the role of donor-advised funds that fund anti-science and anti-vaccine groups.^8^ It’s been reported how some of the private equity firms and investment houses now funnel money into these anti-vaccine groups through their donor-advised funds. So, how do we expose the dark money, a lot of which is also supporting libertarian think tanks or colleges which have become (or are transitioning to the) ultra-right?^9^ We should try to have conversations with these groups to say: Look, dismantling science is not in the best interest of the country. We’re a country that was built on great research institutions and universities, and this is going to weaken us.

Finally, we’ve seen the evaporation of science journalists because newspapers just aren’t hiring them. Popular Science just shut down online,^10^ and National Geographic has laid off a lot of its journalists. Too often, science communication is not seen as a legitimate academic initiative, but, in fact, it’s just as important as getting an NIH grant or bringing in reimbursements for physician services, but universities don’t yet see it that way. So, we’re invisible. I mean, most Americans can’t name a living scientist, and those who can name Bill Nye, The Science Guy, and Neil DeGrasse Tyson. There’s nothing wrong with them and I admire both, but they’re not doing what academics such as many of our Society members typically do, which is to write grants, struggle over major revisions of papers, and present at scientific (for example ASTMH) meetings.

As a result, the American public don’t know how we spend a typical day or what we do on a daily basis, and that’s partly our profession’s fault because we don’t value science and health communication and outreach like we should. So, in engaging the public, we’ve got to consider changing that ecosystem and get the schools of medicine and public health and post-doctoral graduate training programs to realize that science communication is important. Not everyone wants to do it or should be forced to do it, but those who do should have that option. A few years back, I even wrote in a blog about having more science PhDs in K–12 schools because I think they could convey a lot of enthusiasm.

**What’s been your personal experience at Baylor College of Medicine and Texas Children’s Hospital as a science communicator?** Well, I’ve worked with Baylor and Texas Children’s communications staff for 13 years. That’s one thing that’s especially important if you’re going to do public engagement, namely, to develop that relationship with the Office of Communications. You can receive tremendous guidance from professional communications staff. Also, there’s a practical reason: If you’re out there enough in the public domain, eventually you will make a misstep. It’s practically inevitable and happens to most of us. After that happens, you don’t want to be meeting with them for the first time.

Here in Texas, the worry for a lot of these offices of communications is not so much what’s going on nationally but in state government. I wouldn’t know about this unless I spoke with the communications team. The same holds for UCLA where you are based, Claire. It’s a public institution, and you may find out that they’re more concerned about certain California legislators than Washington. So, you need to find out where the sensitive points are and figure out how to navigate them. Also, how we do modern communications, including in science, is rapidly shifting, with new social media platforms, podcasts, streaming, you name it. There are some offices of communications that haven’t adjusted to these new realities or may not realize that the old super risk-averse way of doing business might not work in this new era. In some cases, the old ways leave gaps or a vacuum that could actually enable an anti-science ecosystem to form.

**Have you developed a darker view of humanity in recent years after experiencing physical and psychological threats?** For years, I had a real interest in the history of science and read about scientists’ fate under totalitarian regimes like Stalin’s. I just always thought those things were relegated to the past and never imagined they could happen in 21st century United States. To me, that’s the shocker. So, it’s not so much a darker view of humanity. I thought our darkest days as scientists were behind us, not something that could be so easily resurrected. But I’m still an optimist, developing new vaccines for coronaviruses, neglected diseases, and global health.

What I also worry about now is—if you’re a young person and have lots of choice—is what you decide to do in life when you see scientists hauled in front of C-Span cameras during the Select Subcommittee on the Coronavirus Pandemic hearings, in some cases facing modern day demagogues. Why would I want to become a scientist, right? I mean, it’s hard enough to become a scientist, but now that the profession is being portrayed as public enemies of the state, that’s going to add disincentive. So, I do worry. In the United States, science has had uninterrupted supremacy since the Manhattan Project and the polio vaccine, and the U.S. has been at the forefront in having the greatest universities in the world. Now I worry that era could be shifting – and maybe as a result science will also shift back to Europe or Asia because of the U.S. anti-science agenda, and it will have a terrible impact on our country.

**Are there any thoughts you’d like to share about social media—positive or negative?** Well first, in terms of public engagement, I have a list of the portfolio of things I find meaningful, and it goes way beyond social media. For me writing single-author books is the most fulfilling and has the greatest meaning, and next in line is writing opinion pieces or interviewing on cable news channels or local TV and in certain podcasts. At the bottom of the list is social media. That’s something that younger ASTMH members should think about, because when people think of public engagement, the first thing they think about is social media. I say no: actually, that should be the last thing you think about because it’s now so contaminated and toxic. You do social media sort of as a necessity, but don’t make it the major piece of what you’re doing for public outreach.

**Final question. How can ASTMH members, either individually or collectively, help to counter anti-science? For example, should they write op-eds for their local newspapers or consider other types of public engagement? What do you think about having our Board get behind this?** The most important of course, is to focus on your career to become the best clinician, public health expert, or scientist you can be. Developing a passion for your work and mission is paramount. Also, chart a roadmap to make long-term goals for what problem in life you hope to solve, or describe for yourself and your colleagues what success looks like in 10–15 years. I do a regular whiteboarding exercise for young physicians and scientists along those lines and have been doing this for a couple of decades. It’s very rewarding.

Next, decide if public engagement is important for that road map. It’s not necessarily for everyone, only if you find it meaningful. If you determine that you want a foot in public engagement, think carefully about your impact, and also how to merge it with your professional life. Have discussions with your office of communications and be able to articulate how much of a generalist, versus very specialized in your outreach, you aspire to be. For instance, I have had opportunities to become a more general medical correspondent for websites and cable news networks, but decided I didn’t want to go in that direction. I try instead to focus on my public engagement based on my passions and commitments around global infectious diseases, vaccines, and countering vaccine resistance.

The next is trying to decide what areas of communication to get into. The options now are considerable, everything from books, to websites, opinion magazines, podcasts, local news, cable news channels, streaming services, the sky is the limit. Also, what’s fashionable now may go out of favor in a couple of years, so be flexible and open. Given that emerging infections will continue to be important in the 21st century due to the forces of climate change, urbanization, war and political instability, human and animal migrations, and of course poverty, my own feeling is that ASTMH has an opportunity to be out in front encouraging these opportunities and educating its members about public engagement.

**Thank you, Peter, for this interview and your continuing work on critical issues now facing not just ASTMH, but scientists worldwide.**

## Footnotes

^†^
Initially released in 1935, “*it Can’t Happen Here*” has been described as a “dystopian, political novel” which tells the story of America’s takeover by an authoritarian, dictatorial leader who imposes “totalitarian rule with the help of a ruthless paramilitary force.” Since its publication, it has remained in print and been adapted multiple times.

^‡^
Co-founded by Sir Joseph Rotblat, who received the 1995 Nobel Peace Prize, the Pugwash Conferences were created to bring scientific insight and reason to bear on the catastrophic threat to humanity posed by nuclear and other weapons of mass destruction.

^§^
Heidi J. Larson is an American anthropologist and founding director of the Vaccine Confidence Project. Larson also headed Global Immunisation Communication at UNICEF and authored “*Stuck: How Vaccine Rumors Start and Why They Don’t Go Away*.”

^‖^
Novelli, a professor at Georgetown Business School and leader in social marketing and change, originally co-founded and was President of Porter Novelli, now one of the world’s largest public relations agencies. Over the last 20 years he also served as the CEO of AARP and was a Senior VP at CARE.

^¶^
A prominent physician-executive who served for many years as Executive VP and Chief of Medical Affairs for UnitedHealth Group, Dr. Tuckson has also been appointed to leadership roles at the National Institutes of Health; National Academy of Medicine; and numerous Federal Advisory Committees.

^#^
https://trustinhealthandscience.org/.

** https://twitter.com/COVIDSelect/status/1674501985587056640.
